# Application Effect of ICG Fluorescence Real-Time Imaging Technology in Laparoscopic Hepatectomy

**DOI:** 10.3389/fonc.2022.819960

**Published:** 2022-04-06

**Authors:** Hao Chen, Yumin Wang, Zhiguo Xie, Luyuan Zhang, Yongsheng Ge, Jihai Yu, Chuanhai Zhang, Weidong Jia, Jinliang Ma, Wenbin Liu

**Affiliations:** ^1^Department of Hepatic Surgery, The First Affiliated Hospital of USTC, Division of Life Sciences and Medicine, University of Science and Technology of China, Hefei, China; ^2^Department of Otolaryngology Head and Neck Surgery, Xiangya Hospital, Central South University, Changsha, China; ^3^National Clinical Research Center for Geriatric Disorders, Xiangya Hospital, Changsha, China; ^4^Department of General Surgery, Wannan Medical College, Wuhu, China; ^5^Department of Neurosurgery, First Affiliated Hospital, School of Medicine, Zhejiang University, Hangzhou, China

**Keywords:** laparoscopic hepatectomy, indocyanine green, fluorescence imaging, meta-analysis, hepatocellular carcinoma (HCC)

## Abstract

This study aimed to evaluate the efficiency and safety of indocyanine green (ICG) fluorescence real-time imaging-guided technology in laparoscopic hepatectomy. A retrospective analysis of patients with primary liver cancer in the First Affiliated Hospital of USTC from January 2018 to October 2021, including 48 cases of fluorescence-guided laparoscopic hepatectomy (FGLH) and 60 cases of traditional laparoscopic hepatectomy (LH), was conducted. R0 resection rate, operation time, intraoperative blood loss, complications, hospital stay, and other intraoperative and postoperative indicators of the two groups were analyzed to determine the clinical feasibility and safety of ICG fluorescence real-time imaging-guided technology in laparoscopic hepatectomy. Related databases were searched for retrospective cohort studies and randomized controlled trials comparing FGLH with LH, studies were screened according to preset inclusion and exclusion criteria, literature quality was evaluated, and data were extracted. RevMan 5.3 software was used to conduct a meta-analysis on the extracted data. The results of our clinical data and meta-analysis showed that compared with LH, FGLH increased the R0 resection rate, shortened the operation time and postoperative hospital stay, and reduced blood loss and the occurrence of postoperative complications. Compared with LH, FGLH has a better application effect in laparoscopic hepatectomy, and it is worthy of promotion as it is safe and feasible.

## 1 Introduction

Primary liver cancer is one of the most common malignant tumors of the digestive system. Globally, it ranks sixth in incidence and second in mortality (1). Hepatocellular carcinoma has the characteristics of metastasis along the tumor-bearing portal vein system. Anatomical hepatectomy removes the main tumor and also completely removes the micrometastasis in the tumor-bearing liver segment (2). Even if the resection margin is large enough, there may be residual metastases in the portal vein system of the tumor-bearing liver segment, resulting in early recurrence of non-anatomical hepatectomy (3, 4). Laparoscopic hepatectomy has been widely used in patients with liver tumors, but it is difficult to implement the laparoscopic anatomical hepatectomy procedures advocated by Makuuchi et al. (5) and visually determine the boundaries of liver segments on the monitor. Therefore, the accuracy of laparoscopic anatomical hepatectomy will be affected, but laparoscopic indocyanine green staining of the liver segment can make up for this deficiency (6–8). At present, the safety and effectiveness of indocyanine green (ICG) imaging technology in laparoscopic hepatectomy are still controversial (9, 10). This study retrospectively analyzed the clinical data of 108 patients undergoing laparoscopic hepatectomy from January 2018 to October 2021 in the Department of Hepatic Surgery of the First Affiliated Hospital of the University of Science and Technology of China, integrated relevant clinical studies in recent years to conduct a meta-analysis, and then discussed the safety and application effect of ICG fluorescence real-time imaging-guided technology in laparoscopic hepatectomy.

## 2 Materials and Methods

### 2.1 Clinical Data

#### 2.1.1 General Information

A total of 108 patients who met the inclusion criteria and underwent laparoscopic hepatectomy were recruited from January 2018 to October 2021 in the First Affiliated Hospital of USTC (University of Science and Technology of China). The patients were divided into the experimental group [fluorescence-guided laparoscopic hepatectomy (FGLH)] with 48 cases and the control group [traditional laparoscopic hepatectomy (LH)] with 60 cases. All patients had postoperative pathologically confirmed primary liver cancer, and there was no statistically significant difference in preoperative baseline data between the two groups (**Table 1**). All patients signed an informed consent form before surgery, which complied with medical ethics requirements.

**Table 1:** Preoperative clinical features between the ICG-guided and traditional groups.

Characteristics		ICG group	Traditional group	*t*/*χ*^2^ value	*P*-value
Gender	Male	43	50	0.871	0.351
	Female	5	10		
HBsAg	Positive	40	41	3.200	0.074
	Negative	8	19		
AFP (ng/ml)	≥400	15	23	0.587	0.444
	<400	33	37		
Child–Pugh	A	36	46	0.041	0.840
	B	12	14		
Age (years)		57.3 ± 9.7	56.3 ± 12.1	0.466	0.642

#### 2.1.2 Inclusion Criteria and Exclusion Criteria

According to the indications and contraindications of laparoscopic hepatectomy and ICG fluorescence-guided technology reported in the literature, the following inclusion criteria of laparoscopic hepatectomy were formulated: a) the patient is generally in good condition and important organs do not have serious dysfunction, such as the heart, brain, and lung; b) good liver reserve function; c) no important vascular invasion and venous tumor thrombus; and d) no distant tumor metastasis.

The exclusion criteria were as follows: a) the general condition of the patient is poor and cannot tolerate surgery or long-term pneumoperitoneum, b) allergic to iodine or ICG, c) moderately or severely impaired liver reserve function, d) large tumor and unable to undergo complete laparoscopy resection, e) the tumor is close to the hilum of the liver or invades large blood vessels, f) preoperative imaging shows multiple metastases in the liver or distant metastases, and g) postoperative pathologically confirmed non-liver cancer.

#### 2.1.3 Surgical Methods

**Figure 1:**
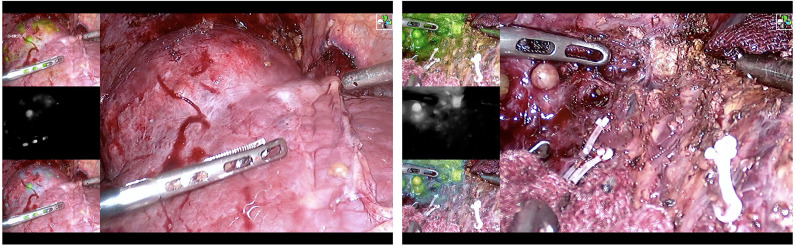
Positive staining. Liver resection under ICG navigation step by step.

Laparoscopic hepatectomy uses the traditional five-hole method. After exposing the Glisson system of the liver segment where the tumor is located, the target liver segment is stained. There are two types of staining methods. a) Positive staining: Under the guidance of percutaneous or laparoscopic ultrasound, the corresponding liver segment or subsegment portal vein is located or the corresponding liver segment or subsegment Glisson is isolated, indocyanine green is injected into the corresponding liver segment or subsegment portal vein (0.25~0.5 mg), and then the stained liver is completely removed (**Figure 1** and **Video S1**). b) Negative staining: After isolating the corresponding liver segment or subsegment Glisson, the corresponding Glisson system is disconnected, indocyanine green (0.75~1.25 mg) is injected through the peripheral vein, and the unstained part of the liver is completely removed. If the portal vein branch of the target liver segment is thin, or the Glisson pedicle of the target liver segment is difficult to expose and the staining fails, intraoperative Doppler ultrasound will be used to define the hepatectomy margin at a distance of 1–2 cm from the tumor boundary. In the process of cutting the liver parenchyma, the ICG fluorescence real-time navigation is used to continuously correct the cut line to ensure sufficient resection margins. All blood vessels and bile ducts encountered along the way should be disconnected reasonably according to the diameter of the pipe. If intraoperative bleeding cannot be controlled, it should be promptly transferred to open surgery.

In the control group, the first hepatic hilum or the liver parenchyma was dissected and the corresponding liver segment or subsegment Glisson system was selected, then clipped and then cut off; the liver parenchyma was cut off along the ischemic line of the liver surface; and the corresponding liver segment was anatomically resected. The Pringle method was used to block the first hepatic portal during the cutting of the liver parenchyma. If an anatomical hepatectomy is not possible, intraoperative color Doppler ultrasound is used to delineate the resection margin of the liver at a distance of 1–2 cm from the tumor boundary to remove the tumor.

#### 2.1.4 Observation Indicators

The operation time, intraoperative blood loss, the number of new lesions found during the operation, and the postoperative liver function [including alanine aminotransferase (ALT), aspartate aminotransferase (AST), total bilirubin (TBIL), and serum albumin (ALB)] and postoperative complications (postoperative bile leakage, bleeding, encapsulated effusion, etc.) were observed and compared.

### 2.2 Meta-Analysis

#### 2.2.1 Literature Search

Using laparoscopic, hepatectomy, indocyanine green, fluorescence, laparoscopic, hepatectomy, and fluorescence as search terms, the CNKI, Wanfang, Weipu, Cochrane Library, PubMed, Embase, and Web of Science databases were searched, and the search time was limited from the establishment of the databases to October 1, 2021. The references of the included documents were retrospectively searched to obtain and supplement the information not found in the search.

#### 2.2.2 Inclusion and Exclusion Criteria

The inclusion criteria were as follows: 1) The subjects included in the study had liver cancer and underwent laparoscopic hepatectomy; 2) the surgical methods included in the study were both ICG fluorescence navigation laparoscopic hepatectomy and traditional laparoscopic hepatectomy, and a comparison of the curative effects of the two surgical methods was conducted; and 3) the outcome indicators of the study include at least one of the following: R0 resection rate, operation time, intraoperative blood loss, postoperative hospital stay, and postoperative complications.

The exclusion criteria were as follows: 1) studies where the full text cannot be obtained or the specific values of the required indicators cannot be obtained; 2) studies that do not include the comparison results of the two surgical methods; 3) repeated publications; and 4) case reports, conference report, and summary or experimental paper.

#### 2.2.3 Data Extraction and Quality Assessment

Two researchers independently screened and read the literature, extracted the data, and scored the quality. In case of inconsistency, a discussion or negotiation together with a third researcher to assist in the solution was conducted. The data extraction content mainly includes the following: first author, year of publication, journal, country, surgical method, and observation indicators. The observation indicators include R0 resection rate, operation time, intraoperative blood loss, postoperative hospital stay, postoperative complications, etc. The Newcastle-Ottawa Scale (NOS) was used to evaluate the quality of the included literature, and NOS score ≥6 was considered high-quality literature.

#### 2.2.4 Statistical Processing

Clinical data were analyzed by statistical package SPSS 16.0 (SPSS Inc., Chicago, IL, USA). Continuous variables were expressed as the mean ± SE and compared using Student’s *t-*test, and non-normally distributed variables were compared using the rank-sum test. Categorical variables were compared using either the *χ*^2^ test or Fisher’s exact test, as deemed appropriate. A *P*-value less than 0.05 was considered as statistically significant.

Meta-analysis was performed using RevMan 5.3 statistical software. Binary variables were analyzed by odds ratio (OR), whereas continuous variables were analyzed by mean difference (MD). In the statistical analysis, 95% confidence interval (CI) was calculated. The heterogeneity between the included studies was analyzed by *χ*^2^ test and determined by *I*^2^ quantitative analysis: if *I*^2^ <50% and *P >*0.05, it is considered that the included studies do not have statistical significance; if *P* ≤0.05 and *I*^2^ ≥50%, it is judged that the included studies have statistical significance. The publication bias of the included literature studies was analyzed using a funnel chart.

## 3 Results

### 3.1 Comparison of Clinicopathological Characteristics Between the Two Groups

There was no significant difference in liver function, platelet, and prothrombin time in the preoperative situation between the two groups (**Table 2**). Considering the general condition and tumor size of the patients, a total of 108 patients were able to tolerate the surgery, and the difference was not statistically significant. The operation time and intraoperative blood loss of the FGLH group were less than those of the LH group (**Table 3**).

**Table 2:** Preoperative liver function index between the ICG-guided and traditional groups.

Characteristics	ICG group	Traditional group	*t*/*χ*^2^ value	*P*-value
ALT	35.6 ± 29.8	33.6 ± 20.7	0.404	0.687
AST	34.1 ± 38.7	32.5 ± 13.9	0.295	0.769
TB	15.8 ± 5.7	16.0 ± 7.2	−0.202	0.840
ALB	43.1 ± 3.3	42.6 ± 3.4	0.797	0.427
PLT	155.9 ± 65.4	155.3 ± 63.7	0.052	0.958
PT	11.5 ± 0.8	11.3 ± 1.1	1.259	0.211

**Table 3:** Intraoperative index between the ICG-guided and traditional groups.

Characteristics	ICG group	Traditional group	*t*/*χ*^2^ value	*P*-value
Tumor size (cm)	4.6 ± 2.5	5.3 ± 2.7	−1.428	0.156
Blood (ml)	307 ± 214	452 ± 401	−2.259	0.026
Operation time (min)	232 ± 61	279 ± 133	−2.253	0.026

The liver function indexes of 48 patients in the FGLH group were significantly better than those of 60 patients in the control group on the first postoperative day, and WBC was lower in the guided group. The incidence of postoperative complications (including postoperative bile leakage, bleeding, and effusion) in the FGLH group was 10/48, compared to 21/60 in the control group. The R0 resection rate in the guided group was 100%, which is higher than the 95.0% (57/60) in the control group, and the postoperative hospital stay was shorter. Fluorescence navigation did not increase the cost, which may be related to the reduction of complications (**Table 4**).

**Table 4:** Postoperative index between the ICG-guided and traditional groups.

Characteristics	ICG group	Traditional group	*t*/*χ*^2^ value	*P*-value
ALT	267.9 ± 135.5	370.7 ± 305.2	−2.338	0.022
AST	260.1 ± 117.1	375.5 ± 317.7	−2.600	0.011
TB	21.4 ± 10.7	22.9 ± 11.5	−0.665	0.508
ALB	36.3 ± 3.4	34.6 ± 3.8	2.357	0.020
WBC	12.4 ± 2.9	13.8 ± 3.3	−2.320	0.022
Hospitalization day	7.6 ± 2.7	8.9 ± 3.5	−2.238	0.027
Cost	40,470 ± 14,790	46,871 ± 51,908	−0.827	0.410

### 3.2 Meta-Analysis

#### 3.2.1 Included Literature

**Figure 2:**
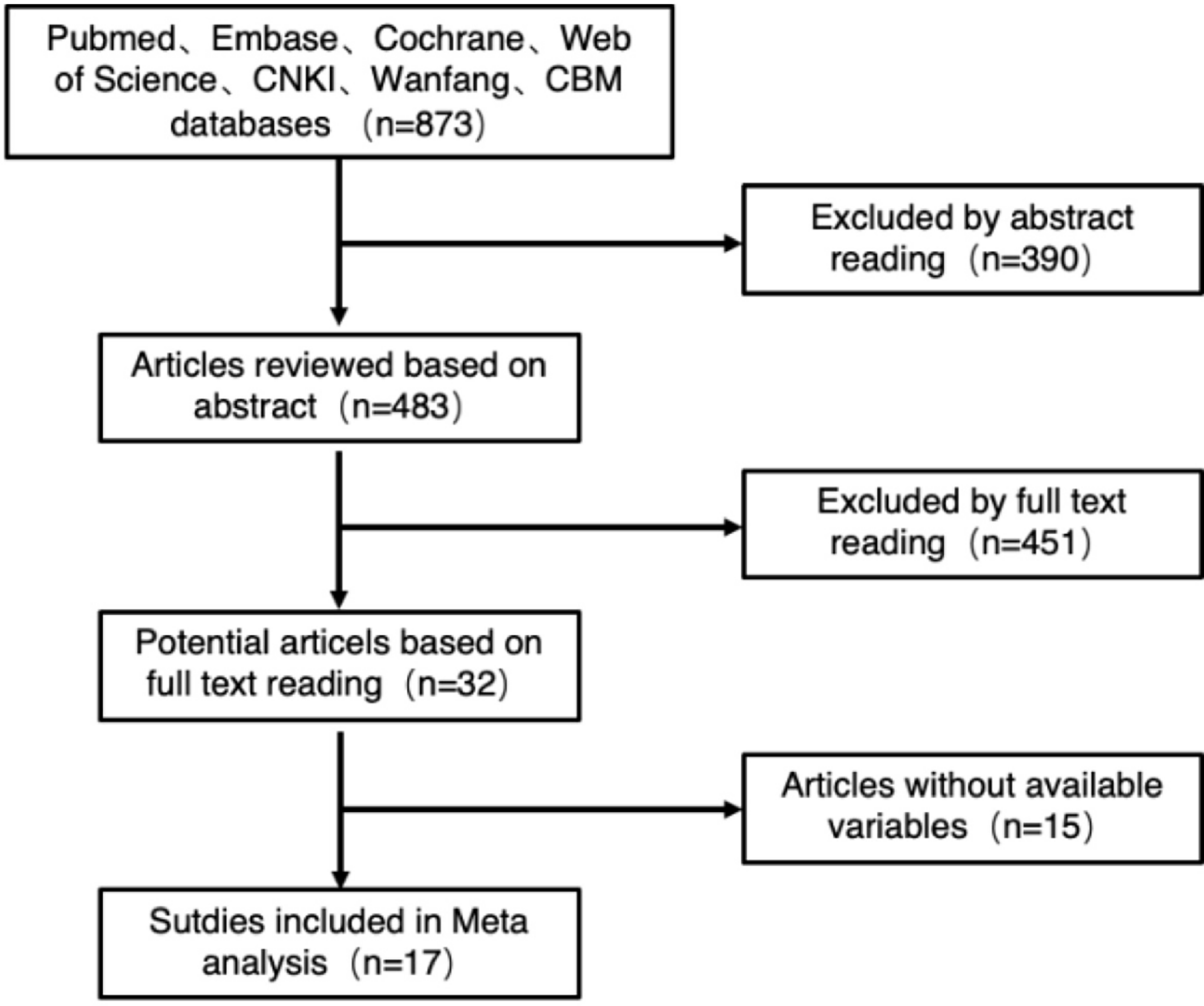
Literature screening flowchart.

A total of 873 related studies from the literature were retrieved, and 17 studies were finally included after deletion and selection, all of which were retrospective cohort studies. The flowchart and the results of literature screening are shown in **Figure 2**. The cumulative sample size was 1,441 cases, consisting of 643 cases in the fluorescence laparoscopy group and 798 cases in the traditional laparoscopy group. The basic information of the included literature is shown in **Table 5**.

**Table 5:** Basic characteristics of the included studies.

Author	Time	Region	ICG group	Traditional group	Characteristics^a^	NOS
Nishino et al. (11)	2017	Japan	23	29	③⑤	8
Aoki et al. (12)	2018	Japan	25	72	②③⑤	8
Chen et al. (13)	2019	China	12	12	①②③④⑤	7
Zhou et al. (14)	2019	China	21	21	①②⑤	8
Xiao et al. (15)	2019	China	67	46	①②⑤	7
Fang et al. (16)	2019	China	23	25	②④	7
Lei et al. (17)	2019	China	36	54	②③⑤	7
Liu et al. (18)	2019	China	24	84	②③④⑤	7
Ma et al. (19)	2019	China	35	40	①②④	7
Lu et al. (20)	2020	China	57	63	①②④	8
Zhang et al. (21)	2020	China	30	34	②③④⑤	8
Pan et al. (22)	2020	China	42	43	①②③④⑤	8
Wang et al. (23)	2020	China	74	74	①⑤	8
Xie and Wu (24)	2020	China	38	65	②③④⑤	7
Zou et al. (25)	2020	China	65	65	②③⑤	7
Wang et al. (26)	2021	China	40	40	①②③④⑤	8
Xin et al. (27)	2021	China	31	31	①②③④⑤	7

a ①, R0 resection; ②, operation time; ③, intraoperative blood loss; ④, postoperative hospital stay; and ⑤, postoperative complications.

#### 3.2.2 Meta-Analysis Results

**Figure 3:**
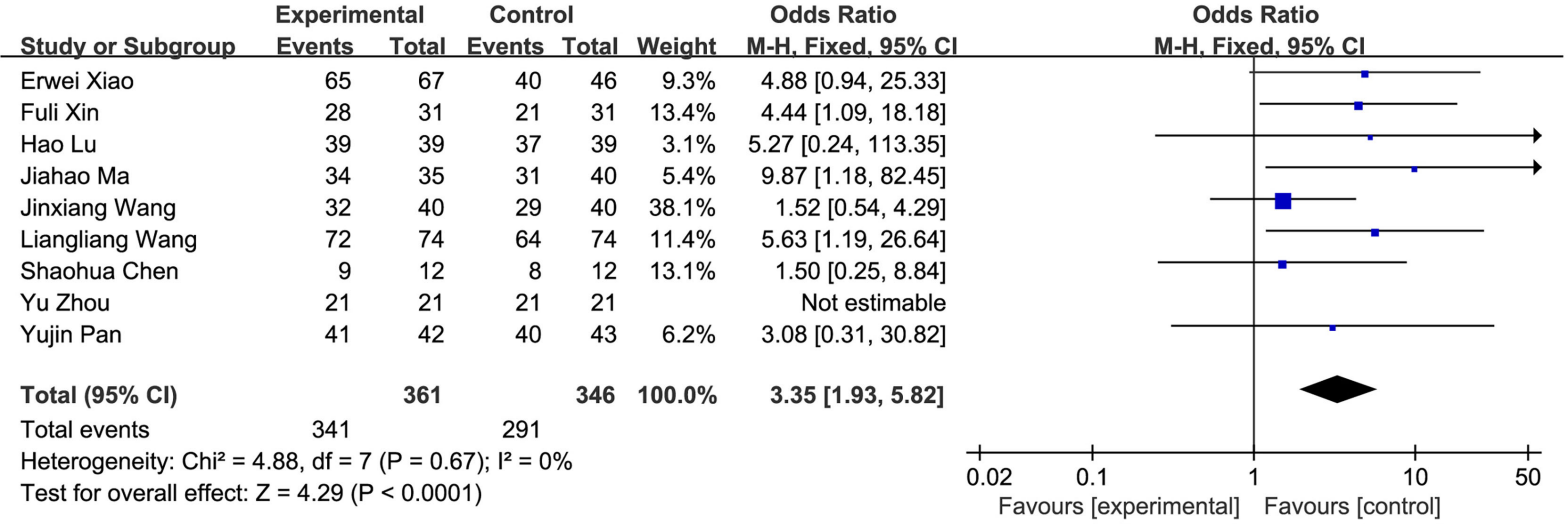
R0 resection between groups with and without ICG fluorescence navigation.

**Figure 4:**
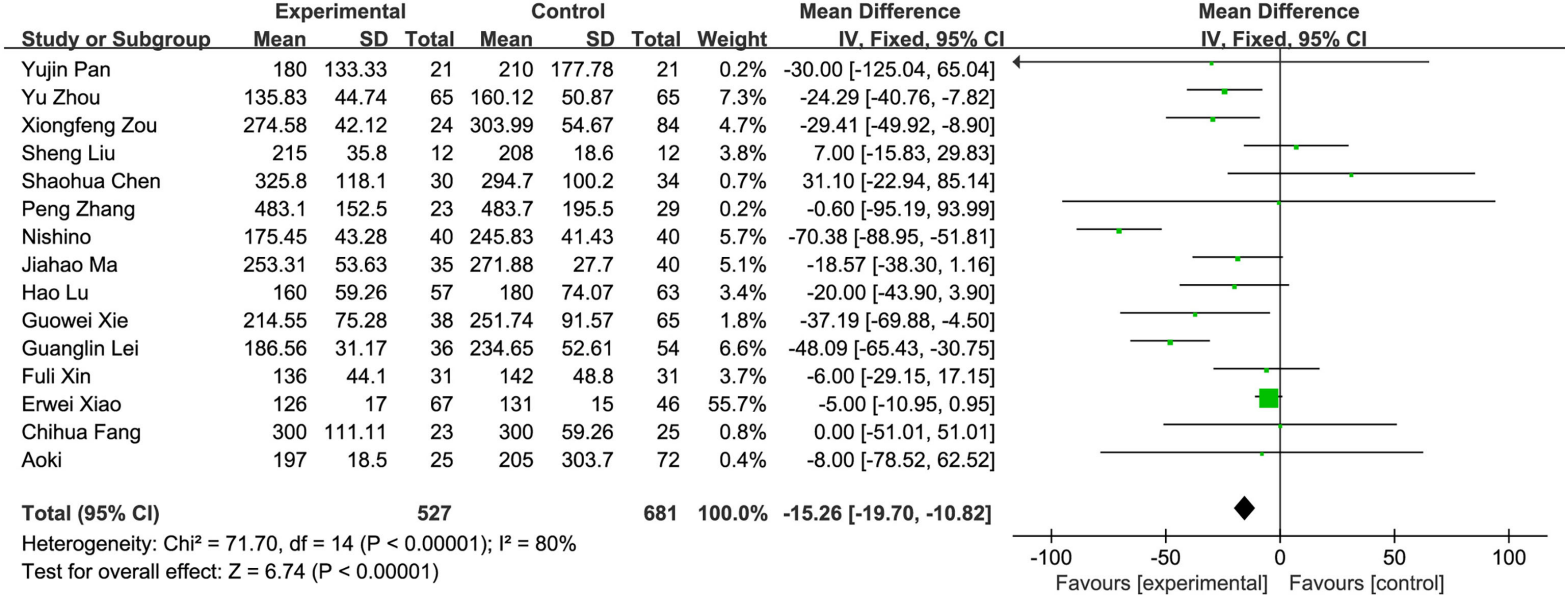
Operation time.

**Figure 5:**
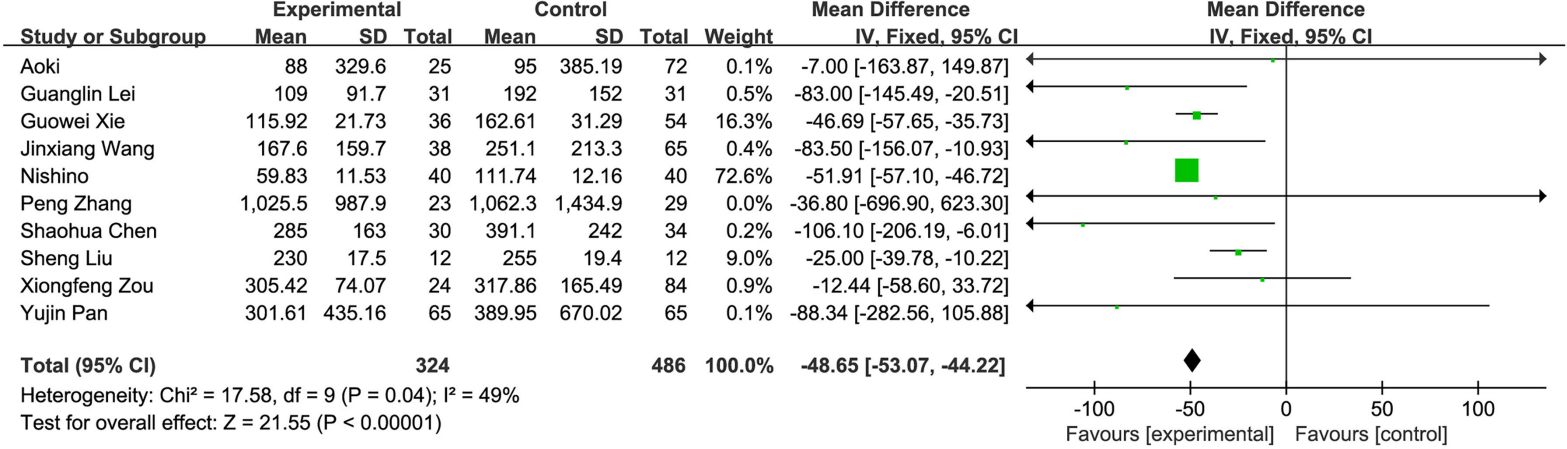
Perioperative bleeding volume.

**Figure 6:**
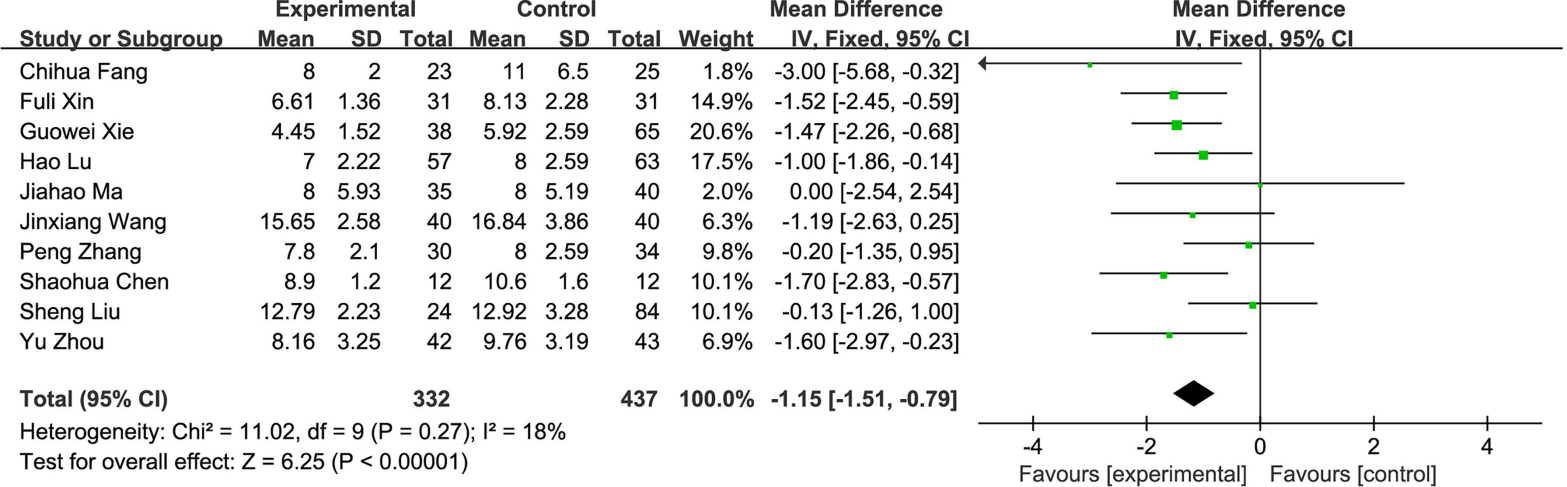
Postoperative hospital stay.

**Figure 7:**
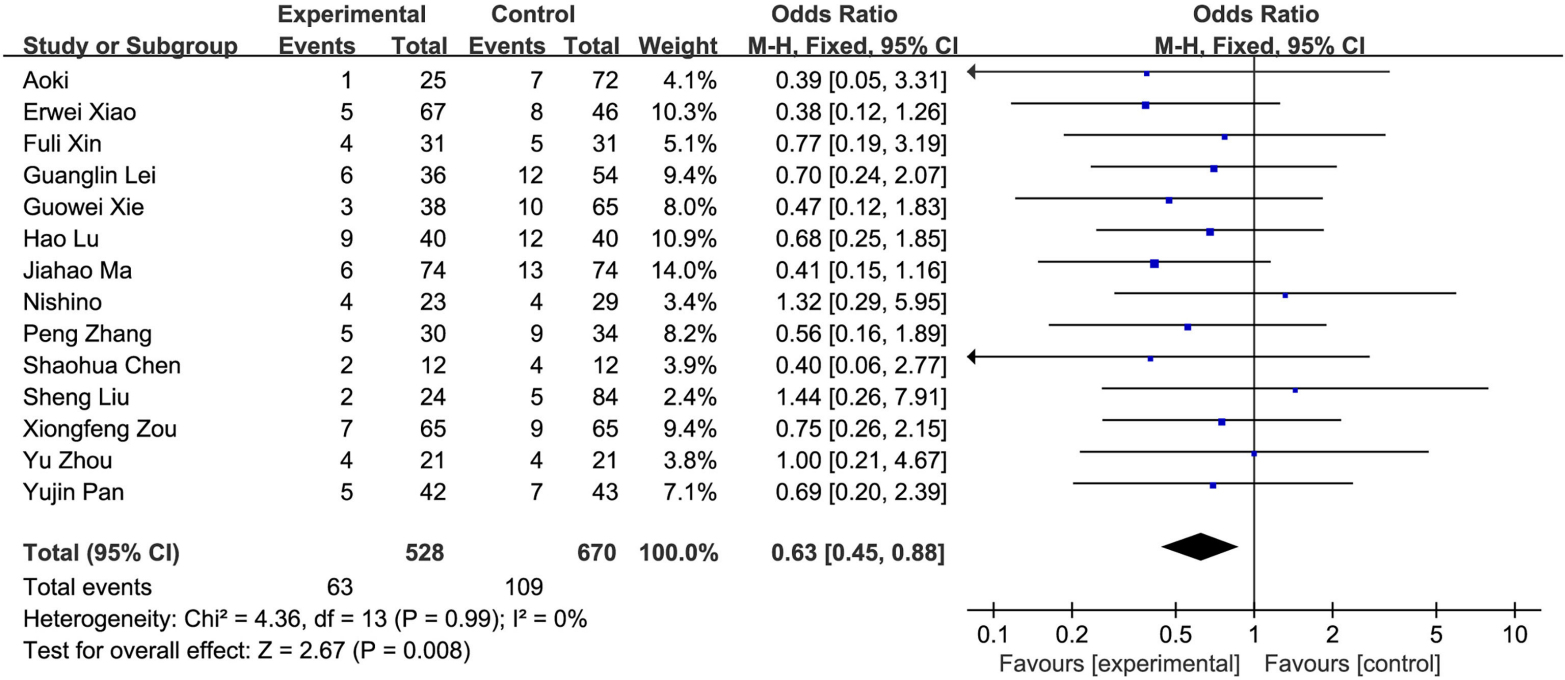
Comparison of postoperative complications.

(1) R0 resection: A total of 9 studies compared the R0 resection rate of the fluorescence laparoscopy group and the traditional laparoscopy group, with a total of 707 cases. There was no heterogeneity among the studies (*I*^2^ = 0%, *P* > 0.05), and the fixed-effects model was used for analysis. The results of the meta-analysis showed that the R0 resection rate between the fluorescence laparoscopy group and the traditional laparoscopy group was significantly different (OR = 3.35, 95% CI: 1.93~5.82, *P* < 0.0001), as shown in **Figure 3**.
(2) Operation time: A total of 15 studies compared the operation time of the fluorescence laparoscopy group and the traditional laparoscopy group, with a total of 1,208 cases. There was heterogeneity among the studies (*I*^2^ = 80%, *P* < 0.05), and the random-effects model was used for analysis. The results of the meta-analysis showed that the difference in operation time between the fluorescence laparoscopy group and the traditional laparoscopy group was statistically significant (MD = −15.26, 95% CI: −19.70~−10.82, *P* < 0.05), as shown in **Figure 4**.
(3) Intraoperative blood loss: A total of 10 studies compared the intraoperative blood loss of the fluorescence laparoscopy group and the traditional laparoscopy group, with a total of 810 cases. The homogeneity of the studies was good (*I*^2^ = 49%, *P* = 0.04), and the fixed-effects model was used for analysis. The results of the meta-analysis showed that intraoperative blood loss between the fluorescence laparoscopy group and the traditional laparoscopy group was significantly different (MD = −48.65, 95% CI: −53.07~−44.22, *P* < 0.00001), as shown in **Figure 5**.
(4) Postoperative hospital stay: A total of 10 studies compared the postoperative hospital stay of the fluorescence laparoscopy group and the traditional laparoscopy group, with a total of 769 cases. The homogeneity of the studies was good (*I*^2^ = 18%, *P* > 0.05), and the fixed-effects model was used for analysis. The results of the meta-analysis showed that the difference in hospital stay between the fluorescence laparoscopy group and the traditional laparoscopy group was statistically significant (MD = −1.15, 95% CI: −1.51~−0.79, *P* < 0.000001), as shown in **Figure 6**.
(5) Postoperative complications: A total of 14 studies compared the postoperative hospital stays of the fluorescence laparoscopy group and the traditional laparoscopy group, with a total of 1,198 cases. There was no heterogeneity among the studies (*I*^2^ = 0%, *P* > 0.05), and the fixed-effects model was used for analysis. The results of the meta-analysis showed that the postoperative complications of the fluorescence laparoscopy group and the traditional laparoscopy group were statistically significant (OR = 0.63, 95% CI: 0.45~0.88, *P* < 0.05), as shown in **Figure 7**.

#### 3.2.3 Publication Bias Analysis

**Figure 8:**
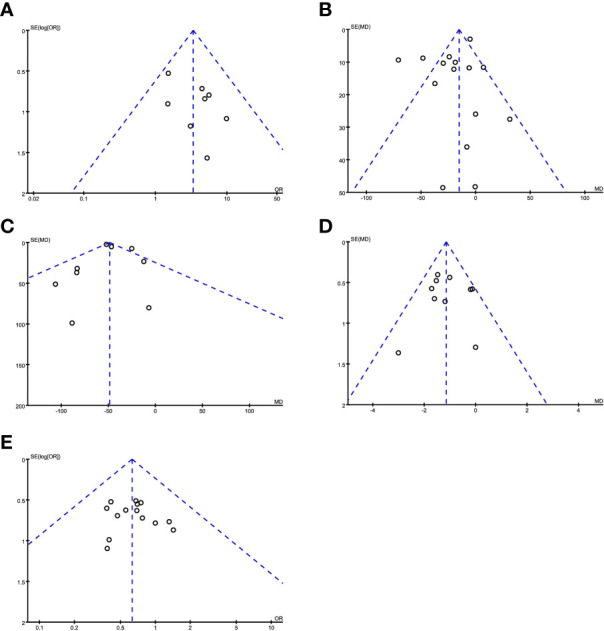
Evaluating publication bias by a funnel chart. **(A)** R0 resection; **(B)** operation time; **(C)** intraoperative blood loss; **(D)** postoperative hospital stay; **(E)** postoperative complications.

Publication bias was evaluated by making a funnel chart. The funnel chart results showed that the scattered points were in the funnel chart and were roughly symmetrically distributed, suggesting that the included studies had no obvious publication bias and had little influence on the results of the meta-analysis (**Figure 8**).

## 4 Discussion

Liver cancer is the sixth most common malignant tumor in the world, and more than half of the newly diagnosed patients are in China (28). Surgical resection is still the best choice for patients with liver cancer (2, 29). No residual tumor (R0) resection of liver tumors is closely related to the prognosis of liver cancer patients. Anatomical hepatectomy is based on the physiological anatomy of the liver and has become the main radical operation for malignant liver tumors, especially tumors in special parts. The core content of the basic concept of precision hepatectomy is to achieve complete eradication of tumors with as little trauma as possible. Therefore, laparoscopic anatomical hepatectomy has become one of the standard treatments for patients with liver cancer.

Laparoscopy technology has the advantages of less trauma and quick recovery (30). At the same time, its unique magnification effect can also assist the surgeon to more clearly identify the tissue structure and various duct systems during the hepatic parenchyma dissection process, improve the efficiency of liver parenchymal dissection, and reduce postoperative complications, thus improving the curative effects and safety of hepatectomy. However, traditional laparoscopic hepatectomy has the following problems: 1) the small tumor cannot be marked in real time during the operation, which makes it difficult to resect multiple small tumors; 2) the resection margin of liver cancer is recommended to be ≥1 cm, and it is difficult to conduct R0 resection by visual observation. 3) During anatomical hepatectomy, it is difficult to adjust the resection plane in real time through the ischemic line, and the liver ducts are not clearly displayed, which may cause complications such as massive bleeding during the operation (31–34). Efficient identification of the boundaries between liver segments is one of the key and difficult points for successful anatomical hepatectomy. At present, the most commonly used method is to ligate the liver pedicle of the target liver segment to form an ischemic line. After the ischemic boundary appears, the liver parenchyma is finely severed. There are obvious limitations in internal discrimination. In addition, the ischemic line is also easily hindered by many factors such as tumor volume, shape, intraoperative bleeding, and tissue scabs, which affect the actual discrimination effect. Therefore, finding a method that can guide and determine the liver segment surface in real time during the entire operation is an important technological breakthrough for laparoscopic anatomical hepatectomy.

ICG is a near-infrared fluorescent dye that can be excited by external light with a wavelength of 750–810 nm, and emits near-infrared light with a wavelength of about 840 nm (35). Because it can be specifically taken up by the liver and excreted through the biliary tract, it does not participate in the characteristics of enterohepatic circulation. Since Ishizawa et al. first reported the application of ICG real-time navigation in laparoscopic hepatectomy in 2009 (36, 37), with the continuous maturity of laparoscopic hepatectomy technology, ICG fluorescence staining technology has gradually been used in laparoscopic anatomical hepatectomy. Injecting ICG to obtain accurate and long-lasting fluorescent staining on the liver surface and parenchyma solves not only the problem of short and easy elution of traditional methylene blue staining but also the problem of the ischemic line caused by adhesions on the liver surface or liver cirrhosis (7, 38, 39). This leads to a clear identification and there is no need to block the hepatic artery. Studies have shown that the success rate of ICG fluorescence staining of liver segments can be as high as 95.8%, which is much higher than the traditional ischemic line marking method of 41.7%, and the effect of ICG fluorescence staining is stable and long-lasting (9).

**Figure 9:**
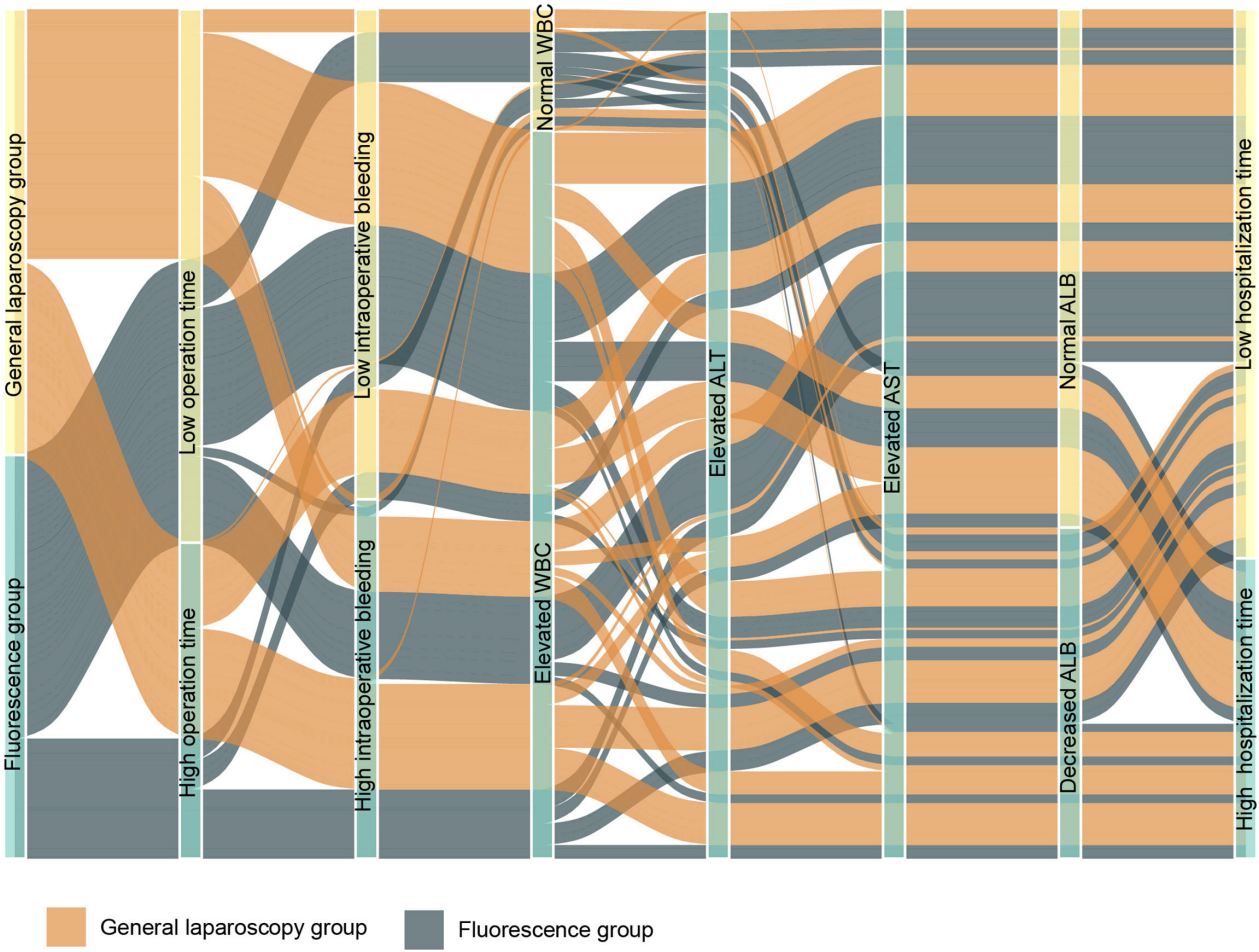
Sankey diagram showing the statistical differences between the two groups.

Our study shows that compared with traditional laparoscopic hepatectomy, the application of ICG real-time navigation to laparoscopic hepatectomy can significantly increase the R0 resection rate, shorten the operation time and postoperative hospital stay, and reduce the intraoperative blood loss and postoperative complications. The main reason for the analysis is that ICG fluorescence staining can clarify the three-dimensional boundary of liver cancer and liver anatomical segmentation, guide the segmentation of liver parenchyma throughout the process, increase the possibility of liver cancer R0 resection, and reduce the repeated positioning of intraoperative ultrasound, which greatly shortens the operation time. Since complete standard anatomical hepatectomy was achieved during the operation, unnecessary damage to important blood vessels in the liver and biliary tracts was avoided, the amount of bleeding during the operation was reduced, and the effective liver tissue volume was greatly preserved, so that the remaining liver was replaced to the greatest extent. Consequently, the liver function index recovers quickly after surgery. An increase in white blood cells indicates that the body’s homeostasis has been destroyed, and can be used to measure the body’s inflammatory response and stress. In this study, the white blood cells of the observation group decreased compared with those of the control group on the first day after the operation, suggesting that ICG fluorescence imaging has a certain effect in reducing the traumatic shock and postoperative stress response of patients. If there is fluorescent leakage in the bile duct during operation, then timely treatment can greatly reduce the postoperative complications (including postoperative bile leakage, pus, and fever caused by encapsulated effusion), reduce the risk of postoperative liver failure, accelerate the postoperative recovery of the patient, and shorten the postoperative hospital stay (**Figure 9**).

ICG molecular fluorescence imaging technology still has defects in the positioning of deep lesions because the penetration depth of fluorescence is about 5–10 mm (40). When ICG fluorescence imaging technology is combined with intraoperative ultrasound, which is difficult to be used for real-time navigation, they can complement each other to achieve a joint effect, and the detection sensitivity can even reach 100%. It has broad application prospects in invasive surgery.

This study also collected relevant literature, systematically evaluated the effectiveness and safety of ICG molecular fluorescence imaging technology in the accurate diagnosis and treatment of liver tumors, and provided reliable evidence-based medical evidence for its widespread clinical promotion. Although this article strictly screened the included literature and formulated the inclusion and exclusion criteria, there were still problems such as the following: 1) the included studies are mostly retrospective cohort studies, and retrospective studies have certain interference factors, which may lead to bias in the research results; 2) different research centers have not performed statistics on the liver segment and pathological stage of the liver cancer patients, so subgroup analysis cannot be performed; 3) most of the literature is for Asian populations and may have an impact on the extrapolation of results; and 4) there is a lack of long-term follow-up data to evaluate long-term efficacy. Therefore, a large sample, multicenter, prospective randomized controlled experiment is still needed to further verify the study.

In the development of ICG imaging-guided techniques for hepatectomy, there is no consensus on the time and dose of preoperative application, and cirrhotic nodules often lead to false positives. The application of ICG dose and timing control requires more clinical data analysis and the development of quantitative indicators to better guide clinical practice. Some scholars believe that intravenous injection of 0.5 mg/kg of ICG within 3 days before surgery can make the lesions show better fluorescence staining during the operation, but some scholars believe that intravenous injection of 7.5 mg of ICG 1 day before surgery also can achieve satisfactory fluorescence display. Our experience is that the preoperative administration time should be adjusted appropriately according to the individual’s degree of cirrhosis: for patients without cirrhosis background, ICG is generally administered intravenously at 0.5 mg/kg 2 to 3 days before surgery; for severe cirrhosis patients, the preoperative injection time can be extended to 7–10 days. Dyeing methods can be divided into positive and negative staining. No matter which staining method is used, the ultimate goal is to distinguish the resected and the retained liver tissue according to the anatomical structure and to guide the operator to better perform anatomical liver resection. Our previous result also suggested that there is no significant difference between positive and reverse stains during liver resection (41).

## 5 Conclusion

ICG molecular fluorescence imaging technology navigation for laparoscopic liver cancer resection is better than conventional laparoscopic hepatectomy, the operation time is shorter, the intraoperative blood loss is less, the surgical trauma is relatively small, the postoperative hospital stay is shorter, and the perioperative operation is better. The long-term benefits are in line with the concept of accelerated rehabilitation surgery, and its safety and effectiveness are worthy of recognition. Future studies need to further demonstrate the impact of this technology on the long-term survival of patients on the basis of more cases and follow-up time. Although this technology is still in the exploratory stage, it has realized the “radical, anatomical, and functional” resection of liver cancer. This minimally invasive and precise technology has brought the treatment of liver cancer into a new era.

## Data Availability Statement

The raw data supporting the conclusions of this article will be made available by the authors, without undue reservation.

## Ethics Statement

The study was approved by the Hospital Ethics Committee (Ethics approval no. 2017 Lunxun No. 114). The patients/participants provided their written informed consent to participate in this study. Written informed consent was obtained from the individual(s) for the publication of any potentially identifiable images or data included in this article.

## Author Contributions

HC, YW, and ZX collected the related data and completed the manuscript and figures. LZ did the statistical analysis. YG, JY, CZ, and WJ did the operations. JM and WL gave constructive guidance and made critical revisions of the manuscript. HC and YW participated in the design of this paper. All authors read and approved the final manuscript.

## Funding

This study was supported by Anhui Provincial Natural Science Foundation (No. 2108085MH285), the Fundamental Research Funds for the Central Universities (No. WK9110000154), Key Research and Development Projects of Anhui Province (201904a07020096, 1804h08020281), Xiangya Hospital Funds for Young Scholar (2020Q13), and China Postdoctoral Science Foundation funded project (2021M693567, 2021TQ0374).

## Conflict of Interest

The authors declare that the research was conducted in the absence of any commercial or financial relationships that could be construed as a potential conflict of interest.

## Publisher’s Note

All claims expressed in this article are solely those of the authors and do not necessarily represent those of their affiliated organizations, or those of the publisher, the editors and the reviewers. Any product that may be evaluated in this article, or claim that may be made by its manufacturer, is not guaranteed or endorsed by the publisher.

## Supplementary Material

The Supplementary Material for this article can be found below:

Supplementary Video 1 An laparoscopic hepatectomy for treatment of a hepatocellular carcinoma patient using ICG fluorescence positive staining navigation is shown. You can download in this link: https://pan.baidu.com/s/1GS4PLg901G6ahW2Ox-CIqA?pwd=8fa3; Code: 8fa3.

## References

[1] Siegel RL Miller KD Jemal A. Cancer Statistics, 2020. CA Cancer J Clin (2020) 70:7–30. doi: 10.3322/caac.21590 31912902

[2] Chidambaranathan-Reghupaty S Fisher PB Sarkar D. Hepatocellular Carcinoma (HCC): Epidemiology, Etiology and Molecular Classification. Adv Cancer Res (2021) 149:1–61. doi: 10.1016/bs.acr.2020.10.001 33579421 PMC8796122

[3] Vogel A Saborowski A. Medical Therapy of HCC. J Hepatol (2021) 76:208–10. doi: 10.1016/j.jhep.2021.05.017 34538616

[4] Liou H Mody K Boyle AW Keaveny AP Croome KP Burns JM. Neoadjuvant Radiation Lobectomy and Immunotherapy for Angioinvasive HCC Resulting in Complete Pathologic Response. Hepatology (2021) 74:525–7. doi: 10.1002/hep.31675 33615518

[5] Eguchi S Kanematsu T Arii S Okazaki M Okita K Omata M. Comparison of the Outcomes Between an Anatomical Subsegmentectomy and a non-Anatomical Minor Hepatectomy for Single Hepatocellular Carcinomas Based on a Japanese Nationwide Survey. Surgery (2008) 143:469–75. doi: 10.1016/j.surg.2007.12.003 18374043

[6] Han HW Shi N Zou YP Zhang YP Lin Y Yin Z. Functional Anatomical Hepatectomy Guided by Indocyanine Green Fluorescence Imaging in Patients With Localized Cholestasis: Report of Four Cases. World J Gastrointest Surg (2021) 13:323–9. doi: 10.4240/wjgs.v13.i3.323 PMC7993002 33796219

[7] Ishizawa T Saiura A Kokudo N. Clinical Application of Indocyanine Green-Fluorescence Imaging During Hepatectomy. Hepatobiliary Surg Nutr (2016) 5:322–8. doi: 10.21037/hbsn.2015.10.01hbsn-05-04-322 PMC4960410 27500144

[8] Kawaguchi Y Nagai M Nomura Y Kokudo N Tanaka N. Usefulness of Indocyanine Green-Fluorescence Imaging During Laparoscopic Hepatectomy to Visualize Subcapsular Hard-to-Identify Hepatic Malignancy. J Surg Oncol (2015) 112:514–6. doi: 10.1002/jso.24021 26345355

[9] Terasawa M Ishizawa T Mise Y Inoue Y Ito H Takahashi Y. Applications of Fusion-Fluorescence Imaging Using Indocyanine Green in Laparoscopic Hepatectomy. Surg Endosc (2017) 31:5111–8. doi: 10.1007/s00464-017-5576-z10.1007/s00464-017-5576-z 28455774

[10] Lwin TM Hoffman RM Bouvet M. Regarding the Applications of Fusion-Fluorescence Imaging Using Indocyanine Green in Laparoscopic Hepatectomy. Transl Gastroenterol Hepatol (2017) 2:70. doi: 10.21037/tgh.2017.08.09tgh-02-2017.08.09 29034343 PMC5638993

[11] Nishino H Hatano E Seo S Nitta T Saito T Nakamura M. Real-Time Navigation for Liver Surgery Using Projection Mapping With Indocyanine Green Fluorescence: Development of the Novel Medical Imaging Projection System. Ann Surg (2018) 267:1134–40. doi: 10.1097/SLA.0000000000002172 28181939

[12] Aoki T Murakami M Koizumi T Matsuda K Fujimori A Kusano T. Determination of the Surgical Margin in Laparoscopic Liver Resections Using Infrared Indocyanine Green Fluorescence. Langenbecks Arch Surg (2018) 403:671–80. doi: 10.1007/s00423-018-1685-y 29915961

[13] Chen SH Zhou L Li XL Shi XJ. Clinical Analysis on Safety and Efficacy of ICG Real-Time Fluorescence Imaging in Laparoscopic Hepatectomy of HCC at Special Location. Med J Chin PLA (2019) 44:74–8. doi: 10.11855/j.issn.0577-7402.2019.04.12

[14] Zhou Y Lin Y Jin H Hou B Yu M Yin Z. Real-Time Navigation Guidance Using Fusion Indocyanine Green Fluorescence Imaging in Laparoscopic Non-Anatomical Hepatectomy of Hepatocellular Carcinomas at Segments 6, 7, or 8 (With Videos). Med Sci Monit (2019) 26:1512–7. doi: 10.12659/MSM.914070 PMC6400019 30806378

[15] Xiao EW Tao LY Wei YK Ma JH Sun XQ Lu YX. Application of Fluorescence-Guided Laparoscopy in Radical Resection of Hepatocellular Carcinoma. Chin J Hepatobiliary Surg (2019) 25:87–9. doi: 10.3760/cma.j.issn.1007-8118.2019.02.002

[16] Fang CH Zhang P Luo HL Zhu W Zeng SL Hu HY. Application of Augmented-Reality Surgical Navigation Technology Combined With ICG Molecular Fluorescence Imaging in Laparoscopic Hepatectomy. Chin J Surg (2019) 57:578–84. doi: 10.3760/cma.j.issn.0529-5815.2019.08.004 31422626

[17] Lei GL Li YY Hu XW Hong ZX. Clinical Application of Indocyanine Green Fluorescence Staining in Laparoscopic Anatomic Hepatectomy in Patients With Hepatocellular Carcinoma and Cirrhosis. J Hepatopancreatobiliary Surg (2019) 31:517–21.

[18] Liu S Yin XM Liu Y Zhu SW Cheng W Shen XB. Safety and Feasibility Analysis of ICG Fluorescence Imaging Laparoscopic Anatomical Right Hepatectomy in the Treatment of Liver Neoplasms. Chin J Pract Surg (2019) 39:944–48. doi: 10.19538/j.cjps.issn1005-2208.2019.09.15

[19] Ma JH Wang LC Wang YF Mu SM Tao LY Li DY. Use of Fusion Indocyanine Green Fluorescence Imaging Technique in Laparoscopic Anatomical Hepatectomy. Chin J Gen Surg (2019) 34:586–89. doi: 10.3760/cma.j.issn.1007-631X.2019.07.009

[20] Lu H Gu J Qian XF Dai XZ. Indocyanine Green Fluorescence Navigation in Laparoscopic Hepatectomy: A Retrospective Single-Center Study of 120 Cases. Surg Today (2021) 51:695–702. doi: 10.1007/s00595-020-02163-8 33128594 PMC8055570

[21] Zhang P Luo H Zhu W Yang J Zeng N Fan Y. Real-Time Navigation for Laparoscopic Hepatectomy Using Image Fusion of Preoperative 3D Surgical Plan and Intraoperative Indocyanine Green Fluorescence Imaging. Surg Endosc (2020) 34:3449–59. doi: 10.1007/s00464-019-07121-1 31705286

[22] Pan YJ Tao LY Sun XQ Lu YX Wang N Yang JH. Application of Indocyanine Green Fluorescence Imaging in Laparoscope Anatomical Hepatectomy. J Abdominal Surg (2020) 33:190–193,199. doi: 10.3969/j.issn.1003-5591.2020.03.006

[23] Wang LL Yang B Zhang WG. Comparison of Fluorescent Laparoscopic Hepatectomy and Conventional Laparoscopic Hepatectomy in the Treatment of Patients With HCC. J Pract Hepatol (2020) 23:427–30. doi: 10.3969/j.issn.1672-5069.2020.03.031

[24] Xie GW Wu H. Application of Using Indocyanine Green Fluorescence Imaging Technology to Open “Four Hepatic Doors”In Laparoscopic Precise Hepatectomy. J Abdominal Surg (2020) 33:184–89. doi: 10.3969/j.issn.1003-5591.2020.03.005

[25] Zou XF Shi N Ruan SY Lin Y Jin HS Wu ZS. Application of Indocyanine Green Fluorescence Imaging in Laparoscopic Hepatectomy. J Abdominal Surg (2020) 33:174–79. doi: 10.3969/j.issn.1003-5591.2020.03.003

[26] Wang JX Li XH Liu TB Lu ZL Zhang J. Application of Indocyanine Green Fluorescence Fusion Imaging Guidance in Laparoscopic Anatomical Liver Resection. Jiangxi Med J (2021) 56:42–44,48. doi: 10.3969/j.issn.1006-2238.2021.01.013

[27] Xin FL Ke Q Liu HZ Wei ZW Liu XL Liu JF. Application of Indocyanine Green Fluorescent Imaging Technique During Laparoscopic Hepatectomy. J Abdominal Surg (2021) 34:271–75,290. doi: 10.3969/j.issn.1003-5591.2021.04.005

[28] Chen W Zheng R Baade PD Zhang S Zeng H Bray F. Cancer Statistics in China, 2015. CA Cancer J Clin (2016) 66:115–32. doi: 10.3322/caac.21338 26808342

[29] Crissien AM Frenette C. Current Management of Hepatocellular Carcinoma. Gastroenterol Hepatol (N Y) (2014) 10:153–61. PMC4014047 24829542

[30] Takamoto T. Improvement and Development in Anatomical Hepatectomy for Hepatocellular Carcinoma. Hepatobiliary Surg Nutr (2021) 10:545–7. doi: 10.21037/hbsn-21-247 PMC8350991 34430540

[31] Liang X Zheng J Xu J Tao L Cai J Liang Y. Laparoscopic Anatomical Portal Territory Hepatectomy Using Glissonean Pedicle Approach (Takasaki Approach) With Indocyanine Green Fluorescence Negative Staining: How I do it. HPB (Oxford) (2021) 23:1392–9. doi: 10.1016/j.hpb.2021.01.014 33593657

[32] Ryu T Honda G Kurata M Kobayashi S Sakamoto K Honjo M. Perioperative and Oncological Outcomes of Laparoscopic Anatomical Hepatectomy for Hepatocellular Carcinoma Introduced Gradually in a Single Center. Surg Endosc (2018) 32:790–8. doi: 10.1007/s00464-017-5745-010.1007/s00464-017-5745-0 28733745

[33] Kim JH. Usefulness of the Ligamentum Venosum as an Anatomical Landmark for Safe Laparoscopic Left Hepatectomy (How I Do it). J Gastrointest Surg (2018) 22:1464–9. doi: 10.1007/s11605-018-3757-210.1007/s11605-018-3757-2 29611092

[34] Tang H Li B Zhang H Dong J Lu W. Comparison of Anatomical and Nonanatomical Hepatectomy for Colorectal Liver Metastasis: A Meta-Analysis of 5207 Patients. Sci Rep (2016) 6:32304. doi: 10.1038/srep32304 27577197 PMC5006087

[35] Ozawa K Fujimoto T Nakatani T Asano M Aoyama H Tobe T. Changes in Hepatic Energy Charge, Blood Ketone Body Ratio, and Indocyanine Green Clearance in Relation to DNA Synthesis After Hepatectomy. Life Sci (1982) 31:647–53. doi: 10.1016/0024-3205(82)90765-2 7132570

[36] Ishizawa T Fukushima N Shibahara J Masuda K Tamura S Aoki T. Real-Time Identification of Liver Cancers by Using Indocyanine Green Fluorescent Imaging. Cancer (2009) 115:2491–504. doi: 10.1002/cncr.24291 19326450

[37] Ishizawa T Bandai Y Kokudo N. Fluorescent Cholangiography Using Indocyanine Green for Laparoscopic Cholecystectomy: An Initial Experience. Arch Surg (2009) 144:381–2. doi: 10.1001/archsurg.2009.9 19380655

[38] Sakoda M Ueno S Iino S Hiwatashi K Minami K Kawasaki Y. Anatomical Laparoscopic Hepatectomy for Hepatocellular Carcinoma Using Indocyanine Green Fluorescence Imaging. J Laparoendosc Adv Surg Tech A (2014) 24:878–82. doi: 10.1089/lap.2014.0243 25347551

[39] Kudo H Ishizawa T Tani K Harada N Ichida A Shimizu A. Visualization of Subcapsular Hepatic Malignancy by Indocyanine-Green Fluorescence Imaging During Laparoscopic Hepatectomy. Surg Endosc (2014) 28:2504–8. doi: 10.1007/s00464-014-3468-z 24566751

[40] Hwang S Ha TY Song GW Jung DH Ahn CS Moon DB. Quantified Risk Assessment for Major Hepatectomy *via* the Indocyanine Green Clearance Rate and Liver Volumetry Combined With Standard Liver Volume. J Gastrointest Surg (2015) 19:1305–14. doi: 10.1007/s11605-015-2846-8 25947549

[41] Yao S Zhang L Ma J Jia W Chen H. Precise Right Hemihepatectomy for the Treatment of Hepatocellular Carcinoma Guided by Fusion ICG Fluorescence Imaging. J Cancer (2020) 11:2465–75. doi: 10.7150/jca.41039 PMC7066014 32201517

